# Maternal autonomy and high-risk pregnancy in Bangladesh: the mediating influences of childbearing practices and antenatal care

**DOI:** 10.1186/s12884-020-03260-9

**Published:** 2020-09-22

**Authors:** Sumaiya Abedin, Dharma Arunachalam

**Affiliations:** 1 Department of Population Science, University of Rajshahi, Rajshahi, Bangladesh; 2 School of Social Sciences, Monash University, Melbourne, Australia

**Keywords:** Autonomy, Antenatal care, Childbearing practices, High-risk pregnancy, Mediating effect

## Abstract

**Background:**

Maternal, infant and neonatal mortality rates are high in Bangladesh. Certain childbearing practices and poor utilisation of antenatal care services make Bangladeshi women more vulnerable to experience poor health during pregnancy and adverse pregnancy outcomes. Also, women in Bangladesh remain considerably subordinate to men in almost all aspects of their lives, from education and work opportunities to healthcare utilisation. This study investigates the severity of health complications during pregnancy in relation to women’s autonomy, and how childbearing practices and utilisation of antenatal care mediate this relationship.

**Methods:**

Data from the most recent Bangladesh Demographic and Health Survey (BDHS) is used in this study. Multinomial regression models (MLRM) are employed to examine the relationship between the outcome variable - high risk pregnancy, and explanatory variables - women’s autonomy, childbearing practices and use of antenatal care.

**Results:**

In Bangladesh, about 41.5% of women experienced high-risk pregnancies involving multiple health complications. Findings showed that women’s autonomy in decision-making, freedom of movement and economic autonomy were significantly associated with high-risk pregnancies. However, women’s autonomy in physical mobility in particular did so only through the mediating factors of maternal childbearing practices and antenatal care. Specifically, both early and delayed childbearing and shorter birth interval increased the likelihood of high-risk (multiple complications) pregnancies by about 30% and 31% respectively, high parity increased the risk by 23% and use of antenatal care decreased it by 46%.

**Conclusions:**

The Women’s decision-making autonomy, freedom of movement and economic autonomy had significant effects on high-risk pregnancies. However, the effects were mediated by both maternal childbearing practices and use of antenatal care in a limited way. Policies and programmes aimed at improving pregnancy outcomes need to focus on all three sets of factors: women’s autonomy, childbearing practices and use of antenatal care.

## Background

Maternal and perinatal deaths as well as poor pregnancy outcomes is recognised as one of the major public health problems in Bangladesh. The common immediate causes of adverse pregnancy outcomes are mainly understood in terms of women’s physiological health during pregnancy and childbirth. Although pregnancy is a universal part of female physiology and biology, this event is shaped by the surrounding social environment [1]. A pregnancy becomes high-risk when either the mother or the newborn (or both) have a significant risk of morbidity or mortality [2, 3]. Studies on pregnancy health and childbirth are mainly concerned with physiological aspects, and only pay limited attention to maternal fertility behaviour, healthcare practices, and women’s socio-economic background. This study extends this knowledge by examining the relationship between maternal autonomy and the severity of pregnancy health complications followed by high-risk pregnancies among Bangladeshi women.

As highlighted in previous studies, women with low levels of education, low income and living in rural areas tend to enjoy low level of autonomy in reproductive health matters which increase the risk of poor pregnancy outcomes [4–9]. The identified risk factors for developing pregnancy complications were also found to be associated with certain childbearing practices which contribute to poor outcomes. A large number of studies have identified the adverse effects of adolescent pregnancies which increased the chance of experiencing several health complications during pregnancy: anaemia, hypertension or pre-eclampsia, gestational diabetes, placental problems, preterm labour and postpartum haemorrhage [10–14]. In Bangladesh, most studies arrived at similar conclusions: adolescent pregnancies hold an increased risk of pregnancy and delivery complications [15–17].

**Fig. 1:**
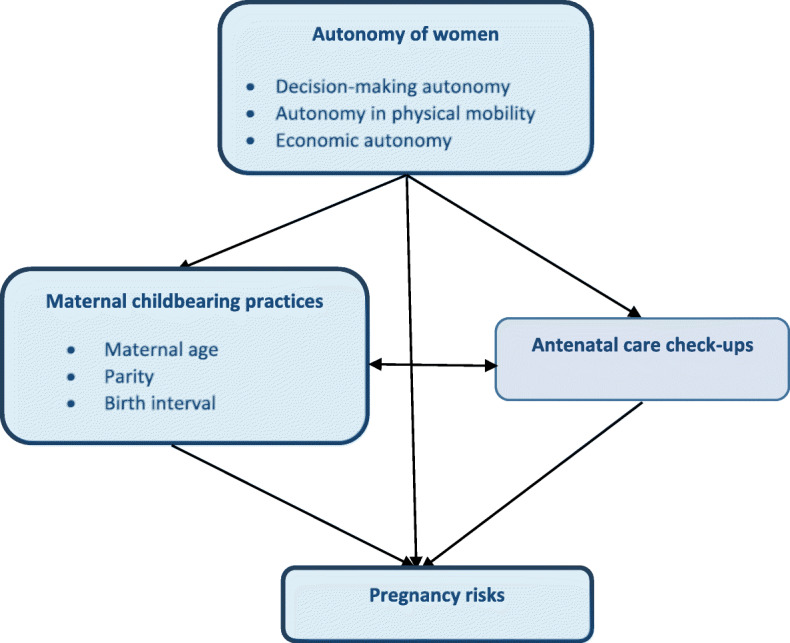
Relationship between autonomy and high-risk pregnancies through the pathways of childbearing practices and antenatal care

Also, women who had given birth to a large number of children (more than three births) and had them in shorter birth intervals of less than 18 months were found to be more vulnerable to experience several pregnancy complications: pre-eclampsia, pregnancy infections, miscarriage, stillbirth and low birth weight [16, 18–22]. Additionally, access to reproductive healthcare services remained a key determinant of a sound pregnancy and healthy outcome [17, 23]. In this context, a large number of studies from developing countries including Bangladesh identified women’s autonomy as a significant factor in the utilisation of antenatal care services [9, 24–27]. Although the associations between pregnancy health status, and maternal childbearing practices as well utilization of maternal healthcare have been widely recognized, the association between maternal autonomy and pregnancy health complications followed by high-risk pregnancies has not yet received similar attention. This study therefore investigates the association between women’s autonomy and high-risk pregnancies, and also examines the mediating role of women’s childbearing practices and antenatal care utilization in this relationship. The assumed key relations between autonomy and high-risk pregnancies through the pathways of maternal childbearing practices and antenatal care are shown in the diagram in Fig. 1.

## Data and methodology

The present study is based on data from the *Verbal Autopsy Questionnaire* of Bangladesh Demographic and Health Survey 2011 (BDHS), which was the most recent survey that used the WHO *Verbal Autopsy Questionnaire*. Through this questionnaire, information on several health complications during pregnancy and delivery was collected from ever-married women aged 10–49 years for their most recent pregnancy in the five years preceding the survey. In the BDHS, the participants were recruited using a two-stage stratified cluster sample of households yielding a response rate of 98%. Details of data collection and management procedures are described elsewhere in 2011 BDHS [28]. The analysis reported here is based on a sample of 327 women for whom information was available for all the variables used in this paper.

### Outcome variable

Any health complication during pregnancy can decipher some degrees of risk, and a number of health complications may decipher higher degrees of risk. A number of complications that a woman may experience during pregnancy are combined by forming a single measure to derive the outcome variable *high-risk-pregnancy*. A score of ‘0’ or ‘1’ is assigned for a complication given that the complication is not experienced or experienced, respectively by a woman during the pregnancy. Scores were assigned to a number of health complications: blunted vision, convulsion, excessive bleeding during labour, gestational diabetes, heart disease, high blood pressure, high fever, long labour, puffy face, severe headache, shortness of breath, and vaginal bleeding. A measure of pregnancy risk is formed by adding scores of all pregnancy complications and this measure ranges from 0 (no complication during pregnancy) to 12 (experiencing all the complications during pregnancy). Based on the univariate distribution of this variable, this variable was collapsed, for analytical purposes, into a three-category outcome variable: no risk (no complication), low risk (one-two complications), and high risk (three or more complications).

### Predictor and mediating variables

The primary predictor variable in this study is comprised of three autonomy variables: *decision-making autonomy* (assessed through questions relating to decisions about household purchases, about their own healthcare, their children’s healthcare, contraception, and the number and timing of births), *autonomy in physical mobility or freedom of movement* (assessed through questions related to the ability of women to visit friends and family, and access to healthcare services without any restriction), and *economic autonomy* (assessed through the ability to spend money when necessary, including for healthcare purposes). Responses to all the items were combined to produce a total score for each type of autonomy. Each total score was then collapsed into three categories: no autonomy (respondent can’t decide at all, either husband or other family member decides about their lives); partial autonomy (respondent has the ability of make joint decisions with husband or other family members); and high autonomy (respondent has the ability to make the decision on their own). As there were relatively few women who reported ‘partial autonomy’, we combined the ‘partial’ and ‘high’ into a single ‘autonomous’ category.

In this study, maternal childbearing practices and uptake of antenatal care services were hypothesized to mediate the associations between maternal autonomy and levels of pregnancy risk. Childbearing practices included three variables: timing of the recent birth (low risk: on-time childbearing, 20 to 34 years; and high risk: early or delayed childbearing, below 20 years or above 34 years), parity (low-risk: 1–3 births; and high-risk: more than three births) and birth interval (low-risk: 18 months and more; and high-risk: less than 18 months). Antenatal care uptake was categorised as: women had insufficient antenatal care (0–2 visits) and had sufficient antenatal care (at least three visits as recommended by the WHO).

### Statistical analysis

The responses obtained from the study population were analysed using descriptive statistics and cross-tabulations. To investigate the influences of maternal autonomy on high-risk pregnancy multinomial logistic regression models (MLRM) were fitted to the categorical response variable -- pregnancy risk, and the odds of low (one or two complications) and high (three or more complications) risks versus no risk were estimated. We fitted two regression models to the data. First, high-risk pregnancy of women is examined with only autonomy variables included in the model, and then the variables maternal childbearing practices and antenatal care were added to the model to assess their roles in influencing the relationship between autonomy and high-risk pregnancy.

## Findings of the study

### Prevalence of pregnancy complications

Table 1 presents information about the most recent live births for which mothers experienced any pregnancy complication. This table shows that over one in ten women (11.5%) had pregnancy-related diabetics. About 9.1% and 6.1% women (i.e. about one in seven women) had experienced two major complications (high blood pressure and convulsion, respectively) that were life-threatening for both the mother and newborn. A significant proportion of women experienced some minor health complications during their pregnancy such as blurred vision (30.3%), severe headache (36.7%), shortness of breath (21.2%) and puffy face (17%). About 28.8% of women had experienced labour lasting more than 12 hours, whereas about 19% women experienced excessive bleeding during labour. When all the complications were combined, it is clear that the vast majority (80%) had experienced at least one complication during pregnancy and childbirth. While about 38.6% of women had experienced one or two complications during pregnancy (low risk), a similar percentage (41.5%) of women were reported to have experienced at least three health complications during pregnancy (high risk).

**Table 1:** Distribution of health complications during pregnancy among Bangladesh women, BDHS 2011 (*n* = 327)

Pregnancy complications	Percentage of women
High blood pressure	9.1
Gestational diabetes	11.5
Heart disease	4.6
Convulsion	6.1
Vaginal discharge	4.5
Puffy face	17.0
Blurred vision	30.3
Severe headache	36.7
High fever	37.1
Long labour	28.8
Excessive bleeding	19.3
Shortness of breath	21.2
**Pregnancy risk**	
** No risk**: No complication	19.9
** Low risk**: One to Two complications	38.6
** High risk**: More than two complications	41.5
** All**	100.0

Table 2 provides information on the three components of autonomy and the four factors, namely maternal age, parity, birth interval and use of antenatal care, that were hypothesised to mediate the relationship between women’s autonomy and the experience of high-risk pregnancy. It is interesting that most Bangladeshi women tended to enjoy at least some degree of autonomy in decision making (61.5%) and freedom of mobility (54.5%). But they seem to enjoy very little economic autonomy: about 86% of women included in the study did not report any degree of autonomy in economic matters. In terms of the mediating factors, the vast majority had their recent birth at the appropriate age (neither early, nor late), have had fewer than four births, and the average interval between births was at least 18 months. Table 2 further shows that one half of the women did not receive sufficient antenatal care.

**Table 2:** Women’s autonomy, maternal childbearing practices and antenatal care among Bangladeshi women, BDHS 2011 (*n* = 327)

Autonomy and mediating factors	Percentage of women
**Autonomy of women**	
* Decision-making autonomy*	
No autonomy	38.5
Autonomous	61.5
* Autonomy in physical mobility*	
No autonomy	45.6
Autonomous	54.4
* Economic autonomy*	
No autonomy	86.2
Autonomous	13.8
**Maternal childbearing practices**	
* Maternal age*	
Low-risk (20 to 34 years)	65.5
High-risk (< 20 years and/or > 34 years)	34.5
* Parity*	
Low-risk (1–3 births)	75.8
High-risk (> 3 births)	24.2
* Birth interval*	
Low-risk (> 18 months)	57.5
High-risk (< 18 months)	42.5
**Antenatal care visits**	
No sufficient (< 3 ANC visits)	49.8
Sufficient (≥ 3 ANC visits)	50.2

### High-risk pregnancy and associated factors

We first examine the relationship between women’s autonomy, mediating factors and high-risk pregnancy through conventional cross-tabulations. We then present the results from the regression analysis. Percentage distribution in Table 3 shows that, of the three dimensions of autonomy, only economic autonomy (ability to spend money when necessary) had a clear association with pregnancy risk although it was not statistically significant (*p* = 0.11). Among those who had the ability to decide on using the available economic resources (e.g. for health care), only 33% reported having experienced multiple complications during their last pregnancy compared to 50% among those who reported that they did not enjoy any autonomy in economic matters. The association was weak (chi-square = 4.36, *p* = 0.11) which may be related to the fact that there was only a relatively small number of respondents who had reported some level of economic autonomy (13.8%).

**Table 3:** Percentage distribution of women by pregnancy risk, and women’s autonomy, maternal childbearing practices and antenatal care of Bangladesh women (*n* = 327)

**Characteristics of respondents**	Pregnancy risk (%)			
	No risk	Low risk	High risk	Chi square (*p*-value)
**Autonomy of women**				
* Decision-making autonomy*				2.874(0.238)
No autonomy	20.6	29.4	50.0	
Autonomous	19.4	34.3	46.3	
* Autonomy in physical mobility*				4.920(0.085)
No autonomy	24.8	28.2	47.0	
Autonomous	15.7	36.0	48.3	
* Economic autonomy*				4.355 (0.113)
No autonomy	19.1	30.9	50.0	
Autonomous	24.4	42.2	33.4	
**Maternal childbearing practices**				
* Maternal age*				12.463 **(0.002)**
Low-risk (20–34 years)	20.9	32.0	47.1	
High-risk (< 20 years and/or > 34 years)	17.6	33.7	49.0	
* Parity*				8.864 **(0.011)**
Low-risk (1–3 births)	21.8	32.7	45.5	
High-risk (> 3 births)	14.0	31.6	54.4	
* Birth interval*				9.034 **(0.012)**
Low-risk (> 18 months)	18.5	30.5	51.0	
High-risk (< 18 months)	21.6	36.2	42.2	
**Antenatal care**				
No sufficient (< 3 ANC visits)	18.4	23.9	57.7	14.342 **(0.001)**
Sufficient (≥ 3 ANC visits)	21.3	40.9	37.8	

Low risk: One-Two pregnancy complications

High-risk: More than two pregnancy complications

In contrast to autonomy variables, all the mediating variables had a strong and statistically significant relationship with pregnancy risks. Early or late childbearing, higher parity and shorter birth intervals increased the risk of experiencing multiple complications (high risk) during pregnancy (Table 3). On the other hand, as would be expected, sufficient utilisation of antenatal health services, decreased the risk of multiple complications.

The estimated odds ratios (OR) from two regression models are presented in Table 4. Model 1 included only the autonomy variables and Model 2 includes both autonomy and mediating variables. In model 1, all three autonomy variables had a strong (OR = 0.63 for economic autonomy) to moderately strong (OR = 0.79 for decision making) association with high risk (multiple complications) pregnancy. When the mediating variables were added in Model 2, women’s autonomy in physical mobility was no more important. This shows that the influence of only one of the three autonomy variables seems to be mediated by factors related to childbearing and the use of antenatal care. However, all mediating variable were still important. Early and late age at childbirth increased the odds of high risk pregnancies by 30%, shorter birth interval and higher parity increased it by 31% and 23% respectively, and sufficient uptake of antenatal care services decreased the odds by almost 46%. The results confirmed that all but one autonomy variable had an independent effect on pregnancy risks, and that pregnancy risks are an outcome of the combined influences of respondents’ childbearing practices, use of antenatal care and women’s autonomy in decision making and economic autonomy. The role of women’s autonomy in pregnancy complications was mediated only in a limited way by childbearing factors and use of antenatal care.

**Table 4:** Estimated odds ratios (multinomial logistic regression models) of the effects of women’s autonomy, maternal childbearing practices, and antenatal care on pregnancy risk of Bangladesh women

**Variables**	Model 1		Model 2	
	**Low-risk** vs. **No risk**	**High-risk** vs. **No risk**	**Low-risk** vs. **No risk**	**High-risk** vs. **No risk**
**Autonomy of women** **Decision-making autonomy**				
No autonomy@	-	-	-	-
Autonomous	0.82	0.79*	0.85	0.81*
**Autonomy in physical mobility**				
No autonomy@	-	-	-	-
Autonomous	0.86*	0.78*	0.71	1.08
**Economic autonomy**				
No autonomy@	-	-	-	-
Autonomous	0.55	0.63*	0.53	0.49*
**Maternal childbearing practicesMaternal age**				
Low-risk (20–34 years)@			-	-
High-risk (< 20 and/or > 34 years)			1.21*	1.30*
**Parity**				
Low-risk (1–3 births)@			-	-
High-risk (> 3 births)			1.24*	1.23*
**Birth interval**				
Low-risk (> 18 months)@			-	-
High-risk (< 18 months)			1.23*	1.31*
**Antenatal care**				
Insufficient (< 3 ANC visits)@			-	-
Sufficient (≥ 3 ANC visits)			0.68*	0.54*

* - *p* < 0.05; @ - reference category

## Discussion

Pregnancy is a complex and long-term biological transition in a woman’s life with many associated health complications. Most pregnancies that result in natural vaginal births involve minor complications, such as nausea and vomiting, headache, blurred vision, puffy face or shortness of breath. On the other hand, major health problems during pregnancy include high blood pressure, convulsion, gestational diabetes, or pregnancy infections. When women experience several of these complications, they are deemed to be carrying a high-risk pregnancy, which contribute to poor outcomes for mother and baby, requiring special medical attention. However, the concept of high-risk pregnancy has not received the same level of attention in the literature as childbearing in general. The present study therefore examined high-risk pregnancies in Bangladesh from a socio-demographic viewpoint.

This study specifies an entrenched pattern of high-risk pregnancies in Bangladesh. The results of the present study identified a significant proportion of women in Bangladesh have experienced a number of health complications during pregnancy. Also, a significant number of women had multiple complications that contributed to an increased chance of pregnancy risks, either low or high. The findings on the relationship between women’s autonomy, childbearing practices and pregnancy risks illustrate that all three dimensions of women’s autonomy, when no other variables were included in the analysis (Table 4, Model 1), had significant influences on high-risk pregnancies. Earlier studies identified some indirect influences of autonomy indicators on pregnancy health status. It was found that freedom to go outside the home for healthcare purposes resulted in better maternal outcomes [9, 26, 29]. Independence in mobility can enable women to gather relevant information about pregnancy health, complications and remedy measures, thus contributed to healthy outcomes. In addition, high level of decision-making power about contraception, having children at a favourable time, spacing and number of children, as well as decision about own and child’s healthcare also contributed to better pregnancy outcomes. In this context, decision-making autonomy, women’s freedom of movement and ability to spend money when necessary to access maternal healthcare facilities reduce the prevalence of several health complications during pregnancy by ensuring higher levels of antenatal care utilization, which in turn decreases the chances of high-risk pregnancy. In this way, all three dimensions of maternal autonomy were found to be associated with sound pregnancy health.

This study explores that the high-risk pregnancy is strongly affected by childbearing practices and the use of antenatal care services. Akin to other developing countries, adolescent pregnancy is prevalent in Bangladesh and is subject to experience more complications. Our study demonstrates that the teenage and late pregnancies are more likely to possess several life-threatening complications. Teenage mothers are more likely to be less educated and less autonomous, and are lacking in awareness and experience regarding the danger signs of pregnancy complications. Thus they are less likely to receive proper prenatal and antenatal care to safeguard themselves from life-threatening complications. At the same time, some health complications during teenage pregnancy are triggered because of the biological effects of early childbearing. This phenomenon has also been focused in some earlier studies [14–16, 30–32]. Our study mimics the exposition of previous research that caesarean deliveries, preterm birth and stillbirths are likely to be more prevalent for grand multiparous (more than three children) women [19, 33]. Our empirical study demonstrates that high-risk birth interval, high birth order, and adolescent or delayed childbearing increase the risk of multiple complications during pregnancy which in turn leads to high-risk pregnancy outcomes.

Earlier studies investigated the adverse effects of short birth intervals on pregnancy health [20, 28, 34]. Women with very short birth intervals experienced anaemia, gestational diabetics and high-blood pressure which contributed increased risks of premature or low birthweight babies. This study also explored that short birth intervals of less than 18 months contributed to multiple health complications during pregnancy, and thus had significant effect on high-risk pregnancies. In addition to this, maternal health complications could occur due to the adverse biological effects of high parity and short birth intervals. Generally, in high order births (more than three) women experience multiple and life-threatening complications. Grand multiparous women are older and less likely to have accessed antenatal care, which results in an increased risk of maternal complications and poor neonatal outcomes [16, 18, 35]. The results also showed a significant relationship between high-risk pregnancies and maternal healthcare utilisation. Only one-half of women in the study had received sufficient antenatal care during pregnancy. Women who received sufficient antenatal care and who received treatment for health complications were more likely to experience sound pregnancy health and reduced chances of high-risk pregnancies.

## Conclusion

The main focus of this research was to investigate the associations between women’s autonomy and high-risk pregnancy, and as discussed above, all three indicators of autonomy appeared to have significant effects on high-risk pregnancy (Model 1). However, once the effects of childbearing practices and antenatal care were accounted for, the effects of autonomy in physical mobility became insignificant. This phenomenon indicates that women’s freedom of movement influences high-risk pregnancies through maternal age, parity, birth interval as well as uptake of antenatal care. Thus, both childbearing practices and antenatal care mediate the association between maternal autonomy in physical mobility and high-risk pregnancies. In other words, the effects of autonomy are mediate only in a limited way through the mediating factors. To reduce the prevalence of high-risk pregnancies in Bangladesh, attention needs to be given to increase the level of autonomy of women, in particular their autonomy in decision making and economic matters. It is equally important to pay attention to women’s age at birth, number of children, intervals between births and access to antenatal care services so as to improve mothers’ reproductive health and pregnancy outcomes for both mother and child.

## Data Availability

The dataset generated and analysed for the present study is freely available at http://dhsprogram.com/data/.

## Abbreviations

ANC: Antenatal care
BDHS: Bangladesh Demographic and Health Survey
WHO: World Health Organization
